# Tissue-Specific Fatty Acids Response to Different Diets in Common Carp (*Cyprinus carpio* L.)

**DOI:** 10.1371/journal.pone.0094759

**Published:** 2014-04-14

**Authors:** Markus Böhm, Sebastian Schultz, Apostolos-Manuel Koussoroplis, Martin J. Kainz

**Affiliations:** 1 WasserCluster –Biologische Station Lunz, Dr. Carl Kupelwieser, Lunz am See, Austria; 2 University of Vienna, Department of Limnology, Wien, Austria; University of Barcelona, Faculty of Biology, Spain

## Abstract

Fish depend on dietary fatty acids (FA) to support their physiological condition and health. Exploring the FA distribution in common carp (*Cyprinus carpio*), one of the world's most consumed freshwater fish, is important to understand how and where FA of different sources are allocated. We investigated diet effects on the composition of polar and neutral lipid fatty acids (PLFA and NLFA, respectively) in eight different tissues (dorsal and ventral muscle, heart, kidney, intestine, eyes, liver and adipose tissue) of common carp. Two-year old carp were exposed to three diet sources (i.e., zooplankton, zooplankton plus supplementary feeds containing vegetable, VO, or fish oil, FO) with different FA composition. The PLFA and NLFA response was clearly tissue-specific after 210 days of feeding on different diets. PLFA were generally rich in omega-3 polyunsaturated FA and only marginally influenced by dietary FA, whereas the NLFA composition strongly reflected dietary FA profiles. However, the NLFA composition in carp tissues varied considerably at low NLFA mass ratios, suggesting that carp is able to regulate the NLFA composition and thus FA quality in its tissues when NLFA contents are low. Finally, this study shows that FO were 3X more retained than VO as NLFA particularly in muscle tissues, indicating that higher nutritional quality feeds are selectively allocated into tissues and thus available for human consumption.

## Introduction

Fatty acids (FA) play a major role in the nutrition of fish [1], [2], [3] and humans [4], [5], [6]. Omega-3 (n-3) and omega-6 (n-6) polyunsaturated FA (PUFA), including eicosapentaenoic (20:5n-3, EPA), docosahexaenoic (22:6n-3, DHA), and arachidonic acid (20:4n-6, ARA) are particularly important for somatic growth, reproduction, and general health of freshwater fish [7], [8]. As is the case for almost all animals, the ability of fish to bioconvert essential precursor PUFA, such as alpha-linolenic (ALA) or linoleic (LIN) acid, is very limited and also depends on dietary supply of target PUFA [9]. Moreover, from a human consumption perspective, the PUFA composition in fish strongly determines their nutritional quality since fish are key in supplying particularly n-3 PUFA to humans [10], [11], [12].

In freshwater fish tissues, FA occur as cell membrane (polar lipid fatty acids, PLFA) and storage lipids (neutral lipid fatty acid, NLFA) [13]. The composition of PLFA affects the physical and biochemical properties of fish cell membranes and therefore their general physiological condition. Cell membrane lipids are particularly rich in long-chain n-3 and n-6 PUFA (LC-PUFA) that are important for maintaining cell membrane fluidity, elasticity, and permeability at colder temperatures [8]. Cell membrane PUFA are also involved in chemical signaling related to immunity, inflammation, mineral balance, and reproductive processes [14], [15]. In contrast, NLFA are stored as long-term energy sources in fish and mobilized during periods of high-energy demand, such as reproduction and migration or during starvation [3]. Results of several studies demonstrate that the NLFA composition in fish usually reflects the FA composition of their diets [16], [17], [18], whereas the PLFA composition in cell membranes is strongly regulated to meet taxa-dependent requirements [19], [3], [18].

Dietary FA have a direct bearing on the FA composition and somatic growth of marine and freshwater fish [8]. However, little is known about dietary FA effects on the FA composition in one of the world's most consumed freshwater fish, common carp (*Cyprinus carpio* L.) [20], [21], [22], [23]. As most studies focus on effects of dietary lipids on the FA composition of edible muscle tissues, there is a lack of knowledge on how dietary FA affect the FA composition of other tissues. Besides a basic physiological interest in learning more about the way FA are retained in different carp tissues, it is relevant to understand how tissues, such as liver, heart, eyes, and intestines that are typically not consumed by humans, retain dietary lipids relative to commonly consumed dorsal and ventral muscle tissues. The scientific rationale of this interest is based on the assumption that some non-edible tissues may accumulate dietary FA more strongly, but are not accessible for human consumption. Understanding how and where dietary FA are allocated within carp may help understand how to design modern aquaculture diets to increase particularly omega-3 FA in dietary muscle tissues and/or how to reuse fish tissues for subsequent feeds rich in omega-3 FA.

We investigated how diet composition influences the retention and subsequently FA composition of polar and neutral lipids in several tissues of farm-raised common carp; i.e., dorsal muscle, ventral muscle, liver, heart, kidney, eyes, intestine and adipose tissue. Assuming that the response of common carp to dietary FA is organ- and lipid class-specific, it was hypothesized that, a) carp regulate their PLFA according to tissue-specific requirements relatively independent of their dietary FA composition (‘quasi homeostasis’), whereas, b) NLFA of all investigated tissues reflect the dietary FA supply and are not tissue-specific. To make results of this study directly applicable to ‘real world’ aquaculture, we designed this study in natural carp ponds rather than fully controlled fish tanks. This study will thus provide detailed information about basic lipid physiology of pond-fed common carp, one of the most important species in freshwater aquaculture worldwide [24], [25].

## Materials and Methods

### Experimental design

Two-year old common carp from the same batch of eggs were initially introduced to and randomly distributed among 3 different aquaculture ponds in temperate Lower Austria (N 48.815049, E 15.297321) to investigate tissue- and lipid class-specific response of FA signatures to different diets. Because fish were not exposed to any dietary threat, harm or experienced any pain or genetic modification it was not required to pass the Ethics Commission. Carp of all ponds had access to natural zooplankton. Carp of pond 1 fed exclusively on zooplankton (N), whereas carp of ponds 2 and 3 were supplied with a supplementary diet of different lipid quality: carp of pond 2 obtained commonly used cereal diet (triticale) enriched with 3% milk thistle (*Silybum marianum*) oil (vegetable oil; VO), while carp of pond 3 were supplemented with a commercially available (GARANT Austria; www.garant.co.at/) compound feed based on marine fishmeal enriched with 18% fish oil (FO; Table 1). Although the latter diet is not typically applied as carp feed, we used this feed to investigate how considerably higher amounts of dietary lipids affects carp and its organs in an effort to elucidate the effect of diet lipid composition on this important freshwater diet fish.

**Table 1:** Relative composition (>0.5%) and ingredients of the used commercial compound feeds containing fish oil (FO; Garant-Tiernahrung, Austria).

Composition	FO
Crude protein	36.0
Total lipids	18.0
Fiber	2.5
Ash	9.0

Ingredients	FO
Soybean meal	25.3
Wheat	13.3
Rapeseed press cake	7.5
Corn	3.5
Peas	5.0
Fish meal (68%)	24.3
Soy beans	5.0
Fish oil	3.0
Fish oil (sprayed on)	10.5
Monocalciumphosphate	1.3
Calcium carbonate	0.5

Feeds were supplied using pendulum feeders (www.alles-fisch.at) that were activated by the fish [26], which allowed us to understand how diet quality, rather than automated supply of feed quantity, affected the FA composition and accumulation in carp. In addition to pond zooplankton as the major diet for carp (see below), supplementary fish feeds were supplied to add VO and FO. The marine compound feed contained a mixture of fishmeal, soybean, wheat, and to a lesser degree rapeseed and corn meal.

### Sampling

To assess the dietary contribution of zooplankton in carp (using stable isotope mixing models; see below), pond zooplankton were sampled using vertical and horizontal net hauls in spring, summer, and fall in each pond. After having been exposed to one of the three different feeds, carp (n = 5 per pond; separately analyzed) of each pond were collected after the cultivation period (210 days; April to November) by professional fishermen, rendered unconscious (blow on the head) and then killed by cardiac incision following the Federal Act on the Protection of Animals, Austria (http://www.ris.bka.gv.at) specifically for this research. All fish were purchased, legally obtained, and analyzed in the lab according to contracts approved for the research project from the Austrian Science Fund (L516-B17). Fish were measured (±0.1 cm) and weighed (±0.1 g), and subsequently samples of eight tissues were taken from each fish (i.e., dorsal and ventral muscle, heart, kidney, intestine, eye balls without the optic nerve after the sclera, liver and visceral adipose tissue) and kept frozen (−80°C) to limit possible lipolytic degradation until further analysis. Zooplankton taxa were identified using a counting chamber (# 435 011; Hydro-bios, Germany) under a microscope.

### Lipid analysis

Total lipids and FA were analyzed as described elsewhere [27]. In brief, homogenized (using mortar and pestle), freeze-dried samples (15–30 mg dry material, DM) were sonicated and vortexed (4X) in a chloroform-methanol (2∶1) mixture. Organic layers were removed and transferred into solvent-rinsed vials. For gravimetrical determination of total lipid mass ratios (i.e., mg lipids g dry weight^−1^), subsamples (100 µL) of the extracts (duplicates) were evaporated and weighed.

Lipid extracts were separated into lipid classes by thin-layer chromatography (TLC). Mass ratios of lipid extracts were adjusted after gravimetry with chloroform to obtain similar lipid amounts (15–25 µg) in the volume (50 µL) applied to the TLC plates for all samples. Polar and neutral lipids were separated by one-dimensional TLC on 10×10 cm silica gel plates (Merck TLC silica gel 60) using hexane:diethylether:methanol:formic acid (90∶20∶3∶2, v/v/v/v) as solvents. After development, plates were sprayed with 0.05% (wt/vol) 8-anilino-4-naphthosulphonic acid in methanol and viewed under UV light to detect lipid fractions. An internal standard (5 µL; nonadecanoic acid in chloroform; 4 mg mL^−1^) was added to each lipid fraction before individual lipid fractions were scraped from the TLC plates and transferred into solvent-rinsed vials.

Fatty acids were derivatized to obtain fatty acid methyl esters (FAME) using toluene and sulfuric acid-methanol-solution (1% v/v, 16 h at 50°C). In contrast to Heissenberger et al. [27], hexane without butylated hydroxytoluene (BHT) was used for each washing step after methylation to avoid possible problems with BHT-related peak interference in chromatograms (data not shown). FAME were identified by comparison with known standards (Supelco37 FAME Mix) using a gas chromatograph (Thermo Scientific TRACE GC Ultra) equipped with a flame ionization detector (FID) and a Supelco SP-2560 column (100 m, 25 mm i.d., 0.2 µm film thickness). Quantification of FA was performed by comparison with a known concentration of the internal standard using Excalibur 1.4 (Thermo Electron Corporation).

### Data analysis

To assess how carp retained FA from different diet sources, data from stable isotope mixing models [26], using δ^13^C and δ^15^N signatures in carp and its diet sources (VO and FO feeds as well as seasonal means of zooplankton), were used to calculate FA accumulation ratios. These mixing models showed that, on average, only 18% of the VO-feeds were retained, whereas FO-feeds by 60% relative to pond zooplankton. Based on these results of diet source retention, we assessed FA accumulation ratios (AR) for carp as:
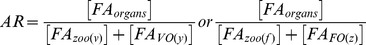
where [FA_organs_] were the FA mass ratios of the investigated carp organs and [FA_zoo(v or f)_], [FA_VO(y)_], and [FA_FO(z)_] were the respective parts of dietary FA mass ratios retained in carp tissues; i.e., FA_zoo(v)_ were 82% of zooplankton FA mass ratios and 18% of FA mass ratios from VO-feeds ([FA_VO(y)_]); whereas FA_zoo(f)_ were 40% of zooplankton FA mass ratios and 60% of FA mass ratios from FO-feeds ([FA_FO(z)_]).

One-way analysis of variance (ANOVA) followed by Tukey's HSD *post-hoc* tests was employed to analyze concentration differences of total lipids and FA among samples. Principal component analyses (PCA) based on arcsin-transformed FA proportions (% of total PLFA or NLFA) were performed separately for PLFA and NLFA to obtain the sample scores (PC_score_) on the first two principal components PC1 and PC2 for each sample. The PC_scores_ were further used for statistical analysis as new variables, representing the major trend in the FA composition [28]. The Pearson correlation coefficient was calculated to relate the PC_score_ to single FA.

Two-way ANOVA were used to assess the effects of tissue and diet on the FA composition (PC_scores_) of the different samples (separately for PLFA and NLFA). The interaction term between the independent variables “tissue” and “diet” of the two-way ANOVA was used to test for tissue-specific NLFA and PLFA response to diet. Data were log-transformed (FA mass ratios per unit biomass) or arcsine-transformed (FA relative proportions) before analysis to meet requirements for normal distribution and homogeneity of variances. Significance level was set at p<0.05. All statistical tests were performed using the XLSTAT software package (version 7.5.2).

## Results

Zooplankton represent the main natural food source for farm-raised common carp in these ponds [29]. In all ponds, the taxonomic composition remained similar with *Daphnia longispina* and *Bosmina longirostis* being the dominant zooplankton species, followed by cyclopoid (*Eucyclops sp.*) and, to a lesser extent, calanoid copepods (*Eudiaptomus sp.*). No benthic invertebrates were observed in sediments (analyses of sediments) or in carp guts (visual inspection of gut contents). While zooplankton were the major diet source in N, results of a previous study on stable isotope analysis (δ^13^C and δ^15^N) in these diets and carp showed that on average only 18% of supplied VO feeds, but 60% of FO were retained in carp [26]. Carp feeding on N and VO were smaller (28±3 cm and 29±2 cm, respectively) and lighter (723±238 g and 655±134 g, respectively) than carp feeding on FO (33±2 cm and 1413±228 g).

### Total lipids

Total lipids in carp (total body contents) exposed to FO-feeds were significantly higher than in carp exposed to N and VO-feeds; the latter did not differ significantly, but varied substantially among carp tissues (Table S1 in File S1). Carp exposed to FO-feeds had the highest total lipid mass ratios in adipose tissues and eyes (770±47and 770±151 mg g^−1^, respectively). Similarly, adipose tissues and eyes had also the highest total lipids in carp exposed to N and VO-feeds, although at significantly lower mass ratios. When compared with carp exposed to N, total lipid mass ratios increased >8X in ventral muscle and >4X in dorsal muscle tissues and eyes in carp exposed to FO-feeds. Liver and intestine total lipid mass ratios were the least affected between carp feeding on N and FO (only 1.6X and 1.5X higher lipid mass ratios, respectively).

### Fatty acid composition

Zooplankton had clearly higher PUFA mass ratios than SAFA and MUFA in all ponds during the entire study period, and consistently more n-3, in particular ALA, SDA, and EPA, than n-6 PUFA. By contrast, supplementary diets had relatively less PUFA, but more MUFA than zooplankton (Table 2). In carp, irrespective of feeding on different diets, palmitic acid (16∶0), stearic acid (18∶0), oleic acid (18:1n-9), ARA, EPA, and DHA were the most abundant FA in PLFA of all tissues (Table S3 in File S1).

**Table 2:** Fatty acid mass ratios (mg g^−1^ dry weight, mean ± (SD), n = 3) of zooplankton (>500 µm) in the three ponds (April-November) and experimental diets: N = natural diet (zooplankton); VO = diet enriched with 3% vegetable oil; FO = diet enriched with 18% marine fish oil; PUFA = polyunsaturated fatty acids.

	Zooplankton						Supplementary diets	
Fatty acids	FO		VO		N		FO	VO
14∶0	5.5	(2.0)	6.0	(2.4)	5.7	(2.1)	9.2	0.7
15∶0	1.0	(0.3)	2.7	(0.4)	0.9	(0.1)	0.7	0.0
16∶0	17.7	(1.8)	15.7	(1.2)	12.6	(1.0)	25.2	4.2
17∶0	1.1	(0.1)	1.7	(0.2)	1.0	(0.1)	0.4	0.0
18∶0	4.9	(0.6)	3.9	(0.6)	3.8	(0.3)	3.9	1.0
20∶0	0.1	(0.1)	0.1	(0.0)	0.2	(0.1)	0.3	0.3
22∶0	0.1	(0.0)	0.2	(0.0)	0.2	(0.0)	0.1	0.3
16:1n-7	4.7	(0.5)	5.0	(0.7)	5.2	(0.4)	8.8	0.0
18:1n-9	6.7	(0.6)	9.8	(1.5)	5.4	(0.3)	22.7	11.7
18:2n-6	4.7	(0.5)	5.8	(0.6)	4.2	(0.4)	9.3	13.5
18:3n-6	0.4	(0.0)	0.9	(0.1)	0.6	(0.0)	0.2	0.0
18:3n-3	13.6	(1.5)	12.9	(1.2)	8.1	(0.5)	2.9	1.3
18:4n-3	19.4	(3.4)	11.4	(1.2)	10.1	(1.0)	5.0	0.0
20:2n-6	1.5	(0.4)	2.4	(1.8)	1.6	(0.9)	0.0	0.0
20:3n-6	0.1	(0.0)	0.2	(0.0)	0.2	(0.0)	0.2	0.0
20:3n-3	0.7	(0.2)	0.2	(0.0)	0.1	(0.0)	0.2	0.0
20:4n-6	2.0	(0.2)	3.5	(0.5)	2.7	(0.2)	1.0	0.0
20:4n-3	3.0	(0.6)	1.4	(0.5)	1.1	(0.1)	1.2	0.0
20:5n-3	17.9	(2.1)	11.7	(1.3)	12.6	(0.8)	13.3	0.0
22:2n-6	0.3	(0.1)	0.1	(0.0)	0.1	(0.0)	0.0	0.0
22:6n-3	9.0	(3.7)	3.5	(1.2)	6.9	(2.3)	14.2	0.0
24:1n-9	0.3	(0.1)	0.3	(0.0)	0.3	(0.1)	1.0	0.0
PUFA	71.0	(7.2)	51.7	(5.1)	46.5	(2.9)	47.7	14.9
n-3 PUFA	62.0	(6.5)	38.8	(5.6)	37.1	(3.0)	36.9	1.3
n-6 PUFA	9.0	(1.0)	12.9	(2.6)	9.4	(1.2)	10.8	13.5

### Fatty acid contents in carp tissues

Polar lipid fatty acids (PLFA; Table S4 in File S1) were generally less retained compared with neutral lipid fatty acids (NLFA; Table S6 in File S1). In general, mostly n-6 C-20 PUFA accumulated in the intestine, kidney, heart, and adipose tissue, but the n-3 PUFA mass ratios of the PLFA fraction in the tissues were less than those of their diets (Table S2 in File S1). PLFA mass ratios were highest in kidneys of carp feeding on pond zooplankton (66.2±3.1 mg g^−1^; Table S3 in File S1) and kidneys had generally the highest mass ratios of total SAFA, MUFA and PUFA. The lowest PUFA mass ratios of all investigated tissues (4.4±0.7 mg g^−1^; FO) were found in eyes. The lowest SAFA mass ratios were detected in dorsal muscle tissue (9.5±0.8 mg g^−1^) of carp exposed to FO diet, whereas the lowest MUFA mass ratios were measured in ventral muscle tissue of carp exposed to VO diet (4.4±0.7 mg g^−1^). Individual PUFA mass ratios differed among the investigated tissues with highest LIN mass ratios measured in the heart of VO-exposed carp (3.3±0.6 mg g^−1^) and highest DHA mass ratios in adipose tissue and kidneys of FO-carp (7.9±2.4 mg g^−1^ and 7.8±1.3 mg g^−1^, respectively). For all tissues, n-6 PUFA mass ratios were lower in FO-exposed carp and the n-3/n-6 ratios were higher in all tissues of FO- (>2) than in N-or VO-exposed carp (<1.7). Consequently, carp exposed to FO diet had significantly higher EPA/ARA ratios than N-or VO-fed fish.

Within NLFA (mainly triacylglycerols, TAG), carp accumulated almost all FA from FO-feeds in the eyes, heart, and muscle tissues. In particular, PUFA were strongly accumulated in eyes and ventral muscle tissue, whereas N- and VO-carp did not accumulate dietary PUFA in their tissues relative to their diets (Table S2 in File S1). The NLFA 16∶0, 18:1n-9, LIN, and ALA were the most abundant FA (Tables S5 and S6 in File S1). Eyes of FO-fed carp had the highest total NLFA mass ratios (661.9±74.1 mg g^−1^), followed by ventral muscle tissue (391.8±33.99 mg g^−1^). Among all tissues, liver tissues generally contained the lowest total NLFA mass ratios independent of diet exposures. Only intestine of FO-carp had lower mean NLFA mass ratios than liver tissues of FO exposed carp. As was the case for PLFA, FO-diet exposure resulted in higher n-3/n-6 ratios in NLFA of all examined tissues than N or VO diets.

### Fatty acid patterns in polar and neutral lipids of common carp

**Figure 1:**
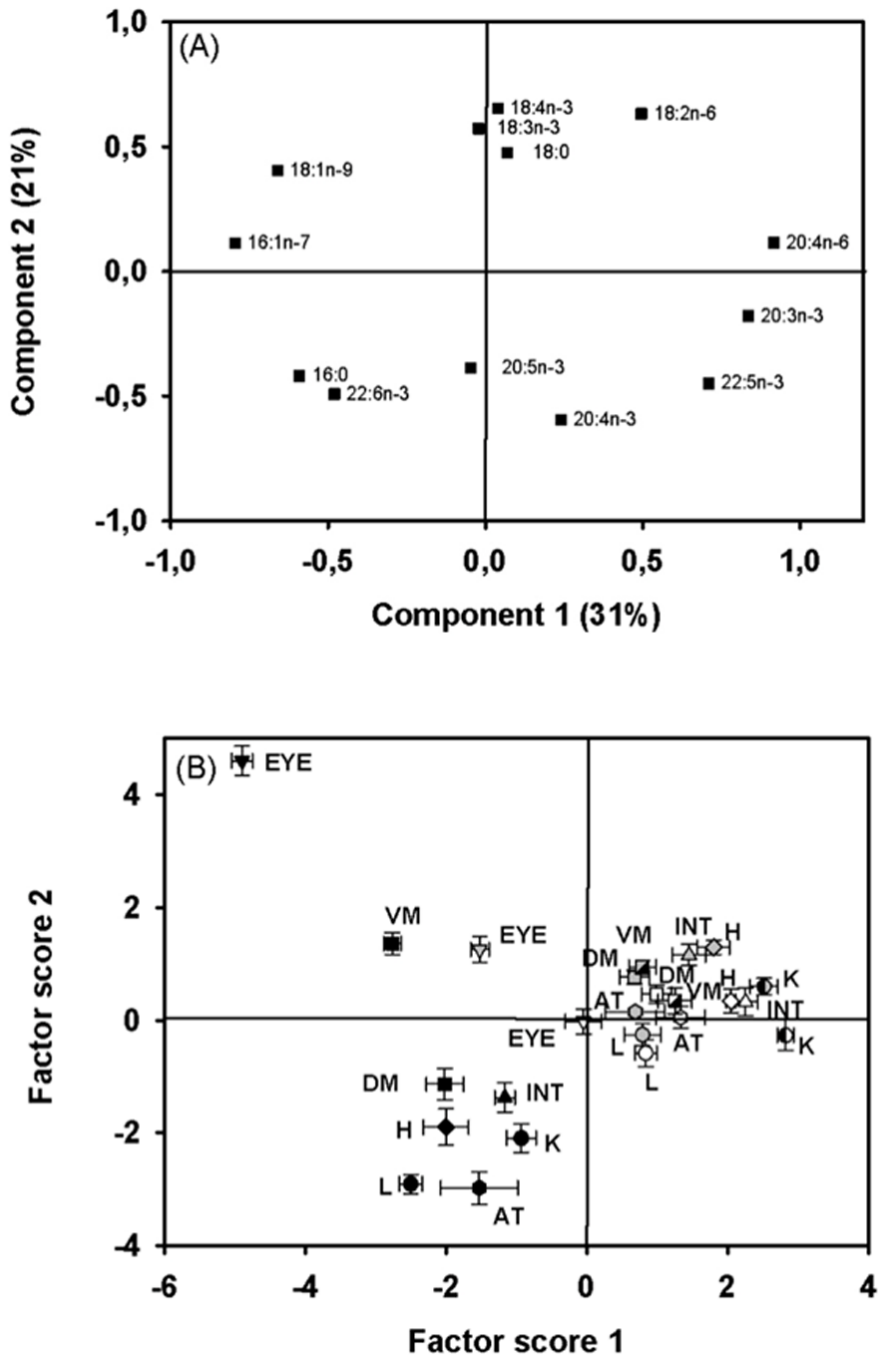
Component plot (A) and factor score plot (B) of the principal component analysis for the fatty acid profile of polar lipids in 8 different tissues of carp fed 3 different diets. White symbols refer to the natural diet (N), grey symbols refer to the vegetable oil (VO) diet and black symbols refer to the fish oil (FO) diet. Tissues are labeled by shortcuts: adipose tissue (AT), dorsal muscle (DM), eyes (EYE), heart (H), intestine (INT), kidney (K), liver (L), and ventral muscle (VM). Data values are represented in the factor score plot as mean ± SEM (n = 5).

Principal component analysis of PLFA (Figure 1) revealed that the first two components (PC1 and PC2) explained 52% of FA eigenvalue variation in carp tissues. The first component (31%) separated fish of the reference (N) and VO pond (positive score), and carp of the FO pond (negative scores) due to positive loadings of n-6 PUFA, 20:3n-3, 20:4n-3, and 22:5n-3and negative loadings of 16∶0, MUFA, and 22:6n-3. The second component accounted for 21% of eigenvalue variation and also separated FO-exposed carp (negative scores with the exception of eyes and ventral muscle tissue) from N and VO carp (positive scores) due to the positive loadings of C_18_ PUFA, 18∶0, and 18:1n-9, and the negative loadings of C_20_ n-3 PUFA and 18∶0. On PC1, there were some consistent patterns among carp tissues to different diet exposure with intestine and kidney showing more positive PC1_score_ (more 16∶0, MUFA, and 22:6n-3) than heart, muscle, liver, adipose tissue, and eyes. The eyes had always the most negative PC1_score_ (more n-6 PUFA, 20:3n-3, 20:4n-3 and 22:5n-3), markedly different from the other tissues. A consistent pattern across diets was also found on PC2 with the eyes having the most positive PC2_scores_ (more C_18_ PUFA, 18∶0 and 18:1n-9), followed by ventral muscle, intestine, dorsal muscle, heart, kidney, adipose tissue and liver. The PC_score_ (PC1 and PC2) of the different samples showed significant correlations with FA proportions used in the PCA, thus justifying its use as a proxy for FA composition in carp (Table 3). The importance of the inter-diet and inter-tissue PLFA composition (PC1_score_ and PC2_score_) was confirmed by the highly significant effect of the factors ‘diet’ and ‘tissue’ (ANOVA; Table 4). Furthermore, the significant interaction term of the ANOVA for both PC1_score_ and PC2_score_ demonstrates that the effect of ‘diet’ on the PLFA composition of ‘tissues’ was different.

**Table 3:** Resulting coefficients of the correlations between the fatty acids (FA) and the principle component scores (PC1 and PC2) for PLFA and NLFA. The asterisks show the level of significance of the correlation coefficients (*p<0.05, **p<0.01, ***p<0.001).

	PLFA				NLFA			
FA	PC1		PC2		PC1		PC2	
16∶0	−0,59	***	−0,43	***	−0,57	***	−0,73	***
16:1n-7	−0,81	***	0,15		0,67	***	−0,57	***
18∶0	0,06		0,48	***	−0,74	***	0,08	
18:1n-9	−0,66	***	0,42	***	−0,58	***	−0,08	
18:2n-6	0,48	***	0,58	***	−0,40	***	0,83	***
18:3n-3	−0,08		0,53	***	0,60	***	0,62	***
18:4n-3	−0,03		0,63	***	0,83	***	−0,23	**
20:3n-3	0,78	***	−0,05		0,59	***	0,52	***
20:4n-6	0,89	***	0,16		0,13		0,58	***
20:4n-3	0,05		−0,49	***	0,88	***	−0,03	
20:5n-3	−0,06		−0,39	***	0,79	***	−0,11	
22:6n-3	−0,48	***	−0,50	***	0,59	***	−0,20	*
22:5n-3	0,71	***	−0,30	***	0,49	***	−0,03	

**Table 4:** Two-way analysis of variance (ANOVA) of the fatty acid composition (principal component 1 & 2 scores) among diet groups, tissues, and the interaction of diet and tissue in polar (PLFA) and neutral lipids (NLFA).

Factors	*df*	*F*	*p*
**PC1 (PLFA)**			
Diet	7	61,5	***
Tissue	2	544,5	***
Interaction	14	2,6	**
**PC2 (PLFA)**			
Diet	7	62,9	***
Tissue	2	92,5	***
Interaction	14	40,7	***
**PC1 (NLFA)**			
Diet	7	8,2	***
Tissue	2	132,0	***
Interaction	14	2,3	*
**PC2 (NLFA)**			
Diet	7	5,3	***
Tissue	2	266,2	***
Interaction	14	1,7	n.s.

Asterisks indicate significant differences: * (*p*<0.05), ** (*p*<0.01), *** (*p*<0.001), n.s.  =  no significant difference (*p*≥0.05).

**Figure 2:**
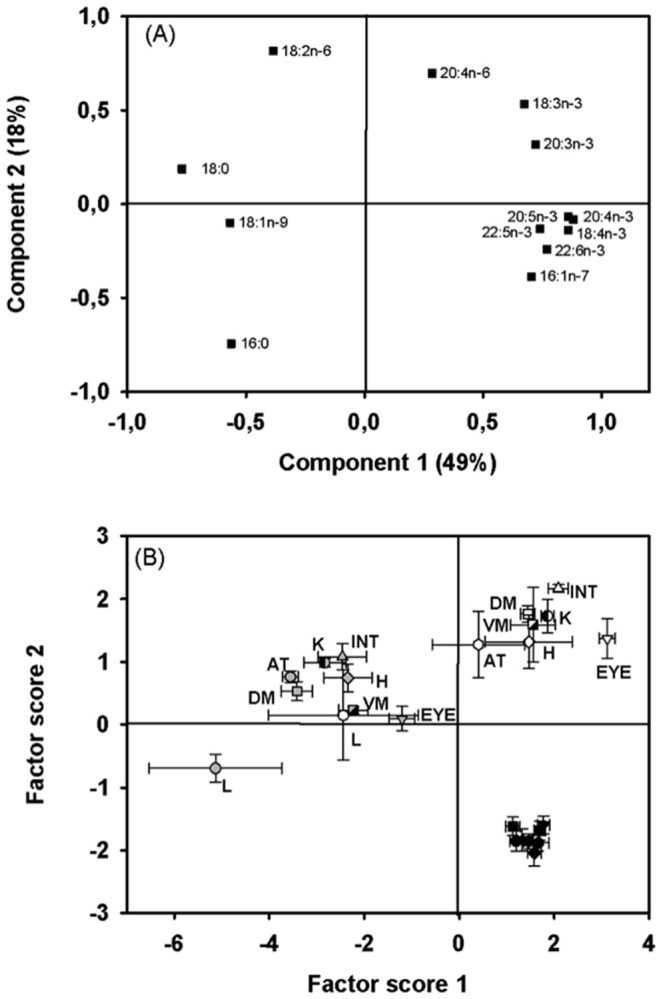
Component plot (A) and factor score plot (B) of the principal component analysis for the fatty acid profile of neutral lipids in 8 different tissues of carp fed 3 different diets. White symbols refer to the natural diet (N), grey symbols refer to the vegetable oil (VO) diet and black symbols refer to the fish oil (FO) diet. Tissues are labeled by shortcuts: adipose tissue (AT), dorsal muscle (DM), eyes (EYE), heart (H), intestine (INT), kidney (K), liver (L), and ventral muscle (VM). Data values are represented in the factor score plot as mean ± SEM (n = 5).

The first two principal components for NLFA explained 67% of the total FA variation (Figure 2): PC1 accounted for 49% of the variation and separated n-3 from n-6 PUFA, SAFA and oleic acid, PC2 (18%) separated n-6 (positive loadings) from most n-3 PUFA (negative loadings). The factor plot revealed three groups on the basis of dietary treatment. Significant differences of tissue FA patterns were measured among diets (p<0.0001) and tissues (p<0.0001) with a significant interaction of these two factors (diet*tissue; p = 0.01). PC1 did not separate FA of diets N and FO (p = 0.545), but PC2 showed that FA of all diet sources were different from each other (p<0.0001). Within diet groups, FA in tissues differed significantly for N (p = 0.001) and VO (p = 0.009), but not significantly in tissues of fish exposed to FO (p = 0.097; Fig. 2B). For fish exposed to FO diet, all tissue FA were highly associated with n-3 PUFA and 16:1n-7 contained in TAG. Liver FA patterns were significantly different from all the other tissues (p = 0.006) and liver FA mass ratios were consistently lower compared to all other tissues.

## Discussion

This study shows how tissue and lipid-class specific FA respond to different diet exposure in common carp. Fatty acids of neutral lipids are tissue specific at low NLFA mass ratios, but represent dietary FA patterns at high NLFA mass ratios. In contrast, the PLFA composition was altered to a far lesser extent, as only when high amounts of n-3 rich fish oil were available, PLFA responded to diet by preferentially incorporating n-3 PUFA in the investigated carp tissues. This tissue-specific FA examination indicates that common carp feeding on zooplankton and/or additional VO-feeds do not accumulate n-3 PUFA or other FA, per unit biomass, relative to their diet, but provides evidence that carp selectively retain 3X more FO-feeds that result in PUFA accumulation mostly in eyes and muscle tissues. This demonstrates that higher quality feeds, as evaluated by PUFA, are not dispersed to tissues equally, but allocated as NLFA to fatty ventral muscle tissues available for human consumption.

### Polar lipid fatty acids (PLFA) in carp

The detected differences in PLFA composition among tissues indicate, confirmed by the highly significant effect of the factor ‘tissue’ (PC1_score_ and PC2_score_), tissue-specific FA requirements of cell membranes in carp. The most marked pattern was the difference between the eyes, the ventral muscle and the rest of the tissues explained by their generally higher proportions of 18:1n-9, 16:1n-7, and 18:3n-3. Although less pronounced between N- and VO-exposed fish, diet had a significant effect on the PLFA composition of the different tissues (PC1_score_ and PC2_score_). Among tissues, observed PLFA changes indicate that membrane FA composition in carp is both, internally regulated and affected to various degrees by diet. The two-way ANOVA detected significant interactive effects on the PLFA composition between tissues and diet (PC_scores_), indicating that inter-tissue PLFA patterns changed with diet. These results suggest that the regulation of PLFA composition varies among tissues, with some tissues being more influenced by dietary FA composition than others.

Based on PCA, C_18_PLFA (except 18:2n-6) differentiated eyes and ventral muscle in carp exposed to FO from the other tissues, which were more influenced by long-chain PUFA such as DHA and EPA. In addition, eyes and ventral muscle tissues showed lower PUFA mass ratios in PL than other tissues, irrespective of their diets. There is evidence from fish feeding studies that eyes of herring [30] and rainbow trout [31] are rich in DHA. Our results show that eyes had generally the highest DHA mass ratios of all tissues, but DHA in eyes varied dramatically with diet exposure. While DHA of PLFA and NLFA was similar when carp was exposed to N or VO diets, carp eyes had lower DHA mass ratios in their polar lipid fraction, but strongly increased DHA as NLFA when fed on and selectively retained (60%) FO diet, demonstrating that additional dietary DHA does not enrich cell membranes (intrinsic regulation), but it allocated and accumulated in storage lipids, and as such available to human consumers.

Fatty acids of polar lipids were similar between carp feeding on N and VO diets. Omega-6 PUFA, mainly LIN and ARA, were present at similarly high mass ratios in PLFA of carp fed on N and VO diets although the supplied VO contained particularly higher LIN than zooplankton. We interpret these similarly high n-6 PUFA because carp only retained 18% of the VO diet. In the presence of an n-3 PUFA-rich diet source (in particular FO), mostly EPA and DHA were preferentially incorporated into the polar lipid fraction at the expense of n-6 PUFA. As expected, PUFA were more efficiently accumulated in cell membranes (PLFA) than SAFA or MUFA (see also [32], [33], [34]) and suggest that carp preferentially retain n-3 and n-6 PUFA as structural lipids even when being exposed to diets that supply less PUFA, which is indicative of general instrinsic PUFA regulation in cell membranes of this or perhaps also other cyprinids.

### Neutral lipid fatty acids in carp

Contrary to our hypothesis, FA in storage lipids were tissue specific in carp feeding on N and VO diets, suggesting that the retention of NLFA is not a simple function of dietary supply. By contrast, and confirming our assumption of tissue specific NLFA response, carp exposed to FO diets largely reflect their dietary FA compositions as shown in NLFA of marine fish [35], [36], [37], [38]. Observed differences in NLFA composition among dietary exposure seem to be linked to the total NFLA mass ratios in different tissues that in turn are presumably related to excess dietary lipids. Total NLFA tissue mass ratios were consistently lower in N- and VO-carp than FO-carp and coincide with total lipid mass ratios in the examined carp tissues. These results suggest that tissue NLFA only track dietary FA when carp tissues are rich in storage lipids.

The NLFA composition results from excess dietary lipids allocated from the liver and deposited as storage lipids [3]. Such mobilized NLFA may differ among tissues as FA binding proteins facilitating the intracellular FA transport are known to be tissue specific [39], [3] and may therefore promote specific NLFA patterns in carp, especially at low neutral lipid mass ratios. Main products of the lipogenesis in fish liver are 16∶0 and 18∶0 and also their desaturated products palmitoleic (16:1n-7) and oleic acid (18:1n-9; [3]). However, dietary n-3 long-chain PUFA effectively reduce lipogenesis [40], [41] and therefore liver FA patterns in carp with dietary access to FO may have been less different from other tissues compared to carp exposed to N and VO, in which lipogenesis likely caused a significant difference in the FA profile compared to all other tissues, as supported by the factor plots (Fig. 2).

Eyes were particularly rich in NLFA as was also reported for fatty fish species such as Atlantic salmon (*Salmo salar*) and rainbow trout (*Oncorhynchus mykiss*) [42]. The low mass ratios of structural PUFA in eyes compared to other tissues was unexpected because fish eyes are generally rich in total PUFA, especially DHA [30]. However, it was also reported that the total amount of PUFA in eyes was higher for lean (<2% fat in muscle tissue) than fatty fish species [42]. Carp feeding on FO diets caused a 4X-6X increase of DHA mass ratios in storage lipids of carp eyes, suggesting organ-specific allocation of excess dietary lipids. By having separated lipid classes of eyes we demonstrate that most of the DHA in carp eyes is associated with storage and less with structural lipids.

In conclusion, the examination of various tissues in carp exposed to different diets indicates the NLFA composition was tissue specific at low TAG mass ratios, but reflected dietary FA composition at high NLFA mass ratios. However, carp changed their PLFA composition by preferentially incorporating long-chain n-3 PUFA when high amounts of dietary fish oil were available. This study increases our overall understanding that commonly applied VO-feeds do not improve the dietary lipid quality of common carp, but the selective retention of PUFA-rich diets results in favorable PUFA accumulation particularly in ventral muscle tissues, but much less in other tissues, which can render the world's mostly consumed freshwater fish an even more important dietary vector of PUFA for humans.

## Supporting Information

File S1 **Table S1. Total lipid mass ratios in tissues of carp after feeding for 210 d on pond zooplankton (N) and zooplankton plus additional feeds containing vegetable oils (VO), and marine fish oils (FO).** **Table S2. Fatty acid accumulation factors calculated as the quotients of, a) PLFA, and, b) NLFA in carp tissues and food sources (N = pond zooplankton; VO = zooplankton and additional feeds containing vegetable oil; FO = zooplankton and additional feeds containing fish oil).** Fatty acids from different food sources in carp were assessed by mixing models using stable isotope analysis of food sources and carp (see text). Fatty acid accumulation in carp tissues is indicated with factors >1 (in bold). **Table S3. Relative (%) polar lipid fatty acid (PLFA) composition of common carp tissues (** ***Cyprinus carpio*** **) exposed to pond zooplankton (N) as well as on additional meals containing vegetable (VO) and fish (FO) oils (% of total FAME, mean ± SD, n = 5).** **Table S4. Polar lipid fatty acid (PLFA) mass ratios (mg g dry weight^−1^) of common carp tissues (** ***Cyprinus carpio*** **) exposed to pond zooplankton (N) as well as to additional meals containing vegetable (VO) and fish (FO) oils (% of total FAME, mean ± SD, n = 5).** **Table S5. Relative (%) neutral lipid fatty acid (NLFA) composition of common carp tissues (** ***Cyprinus carpio*** **) exposed to pond zooplankton (N) as well as to additional meals containing vegetable (VO) and fish (FO) oils (% of total FAME, mean ± SD, n = 5).** **Table S6. Neutral lipid fatty acid (NLFA) mass ratios (mg g dry weight^−1^) of common carp tissues (** ***Cyprinus carpio*** **) exposed to pond zooplankton (N) as well as to additional meals containing vegetable (VO) and fish (FO) oils (% of total FAME, mean ± SD, n = 5).**
(DOCX)
