# Supplementary material for: Tissue-Specific Fatty Acids Response to Different Diets in Common Carp (Cyprinus carpio L.)
Source: PLoS One. 2014 Apr 14;9(4):e94759. doi: 10.1371/journal.pone.0094759 (PMC3986219; doi:10.1371/journal.pone.0094759)

**Supplementary material:**

Table S1

|  | Total lipid mass ratios (± SD; mg g dry weight^-1^) | | |
| --- | --- | --- | --- |
| Carp tissues | N | VO | FO |
| Eye | 183 ± 22 | 333 ± 66 | 770 ± 47 |
| Heart | 107 ± 20 | 143 ± 20 | 287 ± 104 |
| Kidney | 167 ± 25 | 154 ± 22 | 300 ± 74 |
| Liver | 118 ± 11 | 118 ± 11 | 186 ± 52 |
| Dorsal muscle | 46 ± 8 | 48 ± 5 | 200 ± 76 |
| Ventral muscle | 56 ± 4 | 49 ± 6 | 482 ± 64 |
| Adipose tissue | 184 ± 21 | 333 ± 10 | 770 ± 152 |
| Intestine | 152 ± 12 | 127 ± 14 | 221 ± 36 |

Table S2:

a)

|  | Intestine | | | Eye | | | Kidney | | | Liver | | |
| --- | --- | --- | --- | --- | --- | --- | --- | --- | --- | --- | --- | --- |
| PLFA | N | VO | FO | N | VO | FO | N | VO | FO | N | VO | FO |
| 14:0 | 0.1 | 0.1 | 0.1 | 0.0 | 0.1 | 0.1 | 0.1 | 0.1 | 0.2 | 0.1 | 0.0 | 0.1 |
| 15:0 | 1.1 | 0.2 | 0.4 | 0.5 | 0.1 | 0.0 | 1.5 | 0.3 | 0.5 | 1.4 | 0.2 | 0.3 |
| 16:0 | 0.8 | 0.7 | 0.7 | 0.6 | 0.6 | 0.3 | **1.4** | **1.2** | 1.0 | **1.1** | 0.8 | 0.6 |
| 17:0 | **1.5** | 0.5 | 0.6 | 0.6 | 0.2 | 0.0 | 2.0 | 0.7 | 0.6 | 1.2 | 0.4 | 0.3 |
| 18:0 | **1.6** | **1.6** | **1.3** | 1.0 | **1.1** | 0.6 | **1.9** | **2.1** | **1.8** | 1.0 | 1.0 | 0.6 |
| 20:0 | 1.0 | 0.5 | 0.3 | 0.4 | 0.0 | 0.0 | 0.9 | 0.6 | 0.4 | 0.6 | 0.4 | 0.2 |
| 22:0 | 0.4 | 0.2 | 0.0 | 0.0 | 0.0 | 0.0 | 0.5 | 0.4 | 0.0 | 0.2 | 0.1 | 0.0 |
| 16:1n-7 | 0.3 | 0.2 | 0.2 | 0.3 | 0.3 | 0.2 | 0.4 | 0.3 | 0.4 | 0.4 | 0.3 | 0.2 |
| 18:1n-9 | 0.7 | 0.3 | 0.3 | 0.7 | 0.4 | 0.2 | 1.0 | 0.5 | 0.4 | 0.8 | 0.4 | 0.2 |
| 18:2n-6 | 0.5 | 0.2 | 0.1 | 0.3 | 0.2 | 0.1 | 0.7 | 0.4 | 0.2 | 0.4 | 0.2 | 0.1 |
| 18:3n-6 | 0.0 | 0.0 | 0.0 | 0.0 | 0.0 | 0.0 | 0.0 | 0.0 | 0.0 | 0.0 | 0.0 | 0.0 |
| 18:3n-3 | 0.1 | 0.0 | 0.0 | 0.0 | 0.0 | 0.1 | 0.1 | 0.0 | 0.1 | 0.0 | 0.0 | 0.0 |
| 18:4n-3 | 0.1 | 0.0 | 0.0 | 0.0 | 0.0 | 0.0 | 0.1 | 0.1 | 0.0 | 0.0 | 0.0 | 0.0 |
| 20:2n-6 | 0.1 | 0.1 | 0.2 | 0.1 | 0.0 | 0.4 | 0.2 | 0.1 | 0.3 | 0.1 | 0.0 | 0.1 |
| 20:3n-6 | **1.4** | **1.8** | 0.7 | 0.8 | **1.1** | 0.0 | **2.7** | **3.8** | **1.4** | **1.2** | **1.5** | 0.4 |
| 20:3n-3 | **2.4** | 0.6 | 0.2 | 1.0 | 0.0 | 0.0 | **3.4** | **1.2** | 0.4 | **2.1** | 0.6 | 0.1 |
| 20:4n-6 | **2.4** | **1.7** | **1.4** | **1.1** | 0.8 | 0.2 | **4.3** | **3.4** | **2.6** | **2.0** | **1.4** | 0.8 |
| 20:4n-3 | 0.3 | 0.1 | 0.2 | 0.1 | 0.1 | 0.0 | 0.4 | 0.2 | 0.3 | 0.2 | 0.1 | 0.1 |
| 20:5n-3 | 0.3 | 0.2 | 0.2 | 0.1 | 0.1 | 0.1 | 0.4 | 0.3 | 0.4 | 0.2 | 0.1 | 0.1 |
| 22:2n-6 | **1.5** | 1.0 | 1.0 | 0.9 | 1.2 | 0.0 | **2.5** | **1.4** | **1.8** | **1.4** | 0.4 | 0.3 |
| 22:6n-3 | 0.6 | 0.9 | 0.5 | 0.7 | **1.3** | 0.1 | 0.7 | 1.0 | 0.6 | 0.5 | 0.8 | 0.4 |
| 24:1n-9 | 0.3 | 0.4 | 0.2 | 0.2 | 0.0 | 0.0 | 0.5 | 0.8 | 0.4 | 0.1 | 0.2 | 0.1 |
|  |  |  |  |  |  |  |  |  |  |  |  |  |
| PUFA | 0.4 | 0.3 | 0.2 | 0.2 | 0.2 | 0.1 | 0.6 | 0.4 | 0.4 | 0.3 | 0.2 | 0.2 |
| n-3 total | 0.2 | 0.1 | 0.2 | 0.2 | 0.2 | 0.1 | 0.3 | 0.2 | 0.3 | 0.2 | 0.1 | 0.2 |
| n-6 total | 1.0 | 0.5 | 0.3 | 0.5 | 0.3 | 0.1 | **1.6** | **1.1** | 0.6 | 0.8 | 0.4 | 0.2 |

a) cont'd

|  | Dorsal muscle | | | Heart | | | Adipose tissue | | | Ventral muscle | | |
| --- | --- | --- | --- | --- | --- | --- | --- | --- | --- | --- | --- | --- |
| PLFA | N | VO | FO | N | VO | FO | N | VO | FO | N | VO | FO |
| 14:0 | 0.0 | 0.0 | 0.0 | 0.1 | 0.1 | 0.1 | 0.1 | 0.1 | 0.1 | 0.0 | 0.0 | 0.1 |
| 15:0 | 0.5 | 0.1 | 0.2 | 0.9 | 0.3 | 0.4 | 0.9 | 0.2 | 0.4 | 0.4 | 0.1 | 0.0 |
| 16:0 | 0.6 | 0.5 | 0.3 | 0.8 | 1.0 | 0.6 | **1.1** | 1.0 | 0.9 | 0.5 | 0.5 | 0.3 |
| 17:0 | 0.6 | 0.2 | 0.2 | **1.2** | 0.6 | 0.3 | 0.9 | 0.4 | 0.6 | 0.5 | 0.2 | 0.0 |
| 18:0 | 0.9 | 0.8 | 0.5 | **1.3** | **2.2** | **1.1** | **1.4** | **1.4** | **1.5** | 0.7 | 0.8 | 0.6 |
| 20:0 | 0.4 | 0.3 | 0.1 | 0.9 | 1.0 | 0.3 | 0.6 | 0.4 | 0.2 | 0.3 | 0.2 | 0.0 |
| 22:0 | 0.1 | 0.1 | 0.0 | 0.2 | 0.1 | 0.0 | 0.4 | 0.0 | 0.6 | 0.1 | 0.1 | 0.0 |
| 16:1n-7 | 0.2 | 0.2 | 0.2 | 0.3 | 0.3 | 0.2 | 0.2 | 0.3 | 0.2 | 0.2 | 0.2 | 0.2 |
| 18:1n-9 | 0.8 | 0.4 | 0.2 | 0.7 | 0.5 | 0.3 | 0.9 | 0.4 | 0.3 | 0.6 | 0.3 | 0.2 |
| 18:2n-6 | 0.5 | 0.3 | 0.1 | 0.5 | 0.5 | 0.1 | 0.5 | 0.2 | 0.1 | 0.4 | 0.3 | 0.1 |
| 18:3n-6 | 0.0 | 0.1 | 0.0 | 0.0 | 0.0 | 0.0 | 0.0 | 0.0 | 0.0 | 0.0 | 0.1 | 0.0 |
| 18:3n-3 | 0.1 | 0.0 | 0.1 | 0.1 | 0.1 | 0.0 | 0.1 | 0.0 | 0.0 | 0.1 | 0.0 | 0.1 |
| 18:4n-3 | 0.0 | 0.0 | 0.0 | 0.1 | 0.1 | 0.0 | 0.0 | 0.0 | 0.0 | 0.0 | 0.0 | 0.0 |
| 20:2n-6 | 0.1 | 0.0 | 0.1 | 0.1 | 0.1 | 0.2 | 0.1 | 0.1 | 0.2 | 0.1 | 0.0 | 0.3 |
| 20:3n-6 | **1.0** | **1.6** | 0.3 | **1.3** | **2.9** | 0.5 | **2.1** | **2.0** | 0.6 | 0.8 | **1.4** | 0.0 |
| 20:3n-3 | 0.9 | 0.3 | 0.0 | **1.8** | 0.8 | 0.1 | **1.2** | 0.4 | 0.2 | 0.8 | 0.3 | 0.0 |
| 20:4n-6 | **1.1** | 0.9 | 0.5 | **2.2** | **2.6** | **1.3** | **2.7** | **2.2** | **2.1** | 0.9 | 0.8 | 0.4 |
| 20:4n-3 | 0.3 | 0.1 | 0.1 | 0.2 | 0.2 | 0.1 | 0.2 | 0.1 | 0.2 | 0.0 | 0.0 | 0.0 |
| 20:5n-3 | 0.2 | 0.2 | 0.2 | 0.2 | 0.3 | 0.2 | 0.2 | 0.1 | 0.4 | 0.2 | 0.2 | 0.1 |
| 22:2n-6 | **1.4** | 0.7 | 0.7 | **1.2** | **1.4** | **1.2** | **1.2** | 0.9 | **1.7** | **1.1** | 0.4 | 2.0 |
| 22:6n-3 | 0.5 | 1.0 | 0.3 | 0.6 | **1.4** | 0.5 | 0.6 | 1.0 | 0.6 | 0.4 | 0.8 | 0.2 |
| 24:1n-9 | 0.1 | 0.1 | 0.0 | 0.2 | 0.6 | 0.2 | 0.2 | 0.3 | 0.1 | 0.1 | 0.2 | 0.0 |
|  |  |  |  |  |  |  |  |  |  |  |  |  |
| PUFA | 0.3 | 0.2 | 0.1 | 0.3 | 0.4 | 0.2 | 0.4 | 0.3 | 0.3 | 0.2 | 0.2 | 0.1 |
| n-3 total | 0.2 | 0.2 | 0.1 | 0.2 | 0.2 | 0.2 | 0.2 | 0.1 | 0.3 | 0.2 | 0.1 | 0.1 |
| n-6 total | 0.6 | 0.4 | 0.2 | 0.9 | 0.9 | 0.3 | **1.1** | 0.6 | 0.4 | 0.5 | 0.4 | 0.2 |

b)

|  | Intestine | | | Eye | | | Kidney | | | Liver | | |
| --- | --- | --- | --- | --- | --- | --- | --- | --- | --- | --- | --- | --- |
| NLFA | N | VO | FO | N | VO | FO | N | VO | FO | N | VO | FO |
| C14:0 | 0.1 | 0.1 | 0.6 | 0.5 | 1.0 | **6.2** | 0.1 | 0.1 | **1.7** | 0.0 | 0.0 | 0.4 |
| C15:0 | 0.5 | 0.1 | 0.5 | **1.9** | 1.0 | **6.6** | 0.3 | 0.1 | **1.8** | 0.0 | 0.0 | 0.4 |
| C16:0 | 0.3 | 0.2 | 0.7 | 1.0 | **3.8** | **7.4** | 0.2 | 0.2 | **2.2** | 0.0 | 0.0 | 0.5 |
| C17:0 | 0.4 | 0.1 | 0.3 | **1.3** | 0.9 | **3.7** | 0.2 | 0.1 | 1.0 | 0.0 | 0.0 | 0.2 |
| C18:0 | 0.3 | 0.3 | 0.4 | 0.8 | **3.1** | **4.4** | 0.2 | 0.2 | **1.5** | 0.0 | 0.0 | 0.3 |
| C20:0 | 0.3 | 0.2 | 0.0 | 0.7 | **2.6** | **2.1** | 0.1 | 0.3 | 0.7 | 0.0 | 0.0 | 0.1 |
| C22:0 | 0.0 | 0.1 | 0.0 | 0.2 | 0.8 | 0.0 | 0.0 | 0.1 | 0.0 | 0.0 | 0.0 | 0.0 |
| C16:1n-7 | 0.3 | 0.2 | 0.8 | **1.3** | **3.8** | **9.6** | 0.2 | 0.2 | **3.0** | 0.0 | 0.0 | 0.7 |
| C18:1n-9 | 0.7 | 0.4 | 0.7 | **2.7** | **6.4** | **8.3** | 0.4 | 0.4 | **2.6** | 0.1 | 0.0 | 0.6 |
| C18:2n-6 | 0.8 | 0.4 | 0.5 | **3.3** | **5.6** | **6.2** | 0.5 | 0.4 | **1.6** | 0.1 | 0.0 | 0.4 |
| C18:3n-6 | 0.0 | 0.0 | 0.0 | 0.3 | 0.9 | **3.4** | 0.0 | 0.0 | 0.9 | 0.0 | 0.0 | 0.2 |
| C18:3n-3 | 0.3 | 0.0 | 0.3 | 1.0 | 0.7 | **4.5** | 0.2 | 0.0 | 0.9 | 0.0 | 0.0 | 0.2 |
| C18:4n-3 | 0.0 | 0.0 | 0.1 | 0.2 | 0.2 | **1.2** | 0.0 | 0.0 | 0.2 | 0.0 | 0.0 | 0.1 |
| C20:2n-6 | 0.1 | 0.0 | **1.6** | 0.6 | 0.4 | **16.8** | 0.1 | 0.0 | **3.6** | 0.0 | 0.0 | 0.4 |
| C20:3n-6 | 0.4 | 0.2 | 0.3 | **1.7** | **4.1** | **5.1** | 0.0 | 0.2 | **1.4** | 0.0 | 0.0 | 0.3 |
| C20:3n-3 | **1.2** | 0.0 | 0.2 | **3.9** | **1.9** | **3.5** | 0.6 | 0.1 | 0.8 | 0.0 | 0.1 | 0.2 |
| C20:4n-6 | 0.2 | 0.1 | 0.2 | 0.7 | 0.7 | **2.1** | 0.1 | 0.1 | 0.5 | 0.0 | 0.0 | 0.1 |
| C20:4n-3 | 0.2 | 0.0 | 0.2 | 0.6 | 0.5 | **2.0** | 0.1 | 0.0 | 0.5 | 0.0 | 0.0 | 0.2 |
| C20:5n-3 | 0.1 | 0.0 | 0.2 | 0.3 | 0.3 | **2.4** | 0.0 | 0.0 | 0.6 | 0.0 | 0.0 | 0.1 |
| C22:2n-6 | **1.5** | 0.2 | **2.3** | **6.3** | **4.3** | **28.4** | 0.8 | 0.2 | **7.3** | 0.2 | 0.0 | 0.8 |
| C22:6n-3 | 0.0 | 0.0 | 0.2 | 1.0 | **1.2** | **2.0** | 0.0 | 0.0 | 0.5 | 0.0 | 0.0 | 0.1 |
| C24:1n-9 | 0.6 | 0.0 | 0.1 | 0.0 | 0.0 | 0.5 | 0.7 | **3.6** | 0.3 | 0.0 | 0.0 | 0.1 |
|  |  |  |  |  |  |  |  |  |  |  |  |  |
| PUFA | 0.2 | 0.1 | 0.2 | 0.8 | 1.3 | **3.0** | 0.1 | 0.1 | 0.7 | 0.0 | 0.0 | 0.2 |
| n-3 total | 0.1 | 0.0 | 0.2 | 0.6 | 0.5 | **2.3** | 0.1 | 0.0 | 0.5 | 0.0 | 0.0 | 0.1 |
| n-6 total | 0.5 | 0.2 | 0.6 | **1.9** | **3.4** | **6.4** | 0.3 | 0.3 | **1.6** | 0.0 | 0.0 | 0.3 |

b) cont'd

|  | Dorsal muscle | | | Heart | | | Adipose tissue | | | Ventral muscle | | |
| --- | --- | --- | --- | --- | --- | --- | --- | --- | --- | --- | --- | --- |
| NLFA | N | VO | FO | N | VO | FO | N | VO | FO | N | VO | FO |
| 14:0 | 0.0 | 0.0 | **1.7** | 0.0 | 0.1 | **1.9** | 0.0 | 0.0 | 0.9 | 0.0 | 0.0 | **3.3** |
| 15:0 | 0.1 | 0.0 | **1.9** | 0.2 | 0.1 | **2.1** | 0.1 | 0.0 | 0.9 | 0.2 | 0.0 | **3.5** |
| 16:0 | 0.1 | 0.1 | **2.1** | 0.1 | 0.5 | **2.4** | 0.0 | 0.1 | **1.2** | 0.1 | 0.1 | **4.2** |
| 17:0 | 0.1 | 0.0 | **1.1** | 0.2 | 0.1 | **1.1** | 0.0 | 0.0 | 0.6 | 0.1 | 0.0 | **2.3** |
| 18:0 | 0.1 | 0.1 | **1.4** | 0.1 | 0.5 | **1.5** | 0.0 | 0.1 | 0.8 | 0.1 | 0.2 | **2.9** |
| 20:0 | 0.1 | 0.0 | 0.7 | 0.0 | 0.4 | 0.6 | 0.0 | 0.1 | 0.2 | 0.1 | 0.1 | **1.5** |
| 22:0 | 0.0 | 0.0 | 0.0 | 0.0 | 0.1 | 0.0 | 0.0 | 0.0 | 0.3 | 0.1 | 0.0 | 0.0 |
| 16:1n-7 | 0.1 | 0.1 | **2.7** | 0.1 | 0.4 | **3.3** | 0.0 | 0.1 | **1.7** | 0.1 | 0.1 | **5.8** |
| 18:1n-9 | 0.2 | 0.1 | **2.4** | 0.2 | 0.8 | **2.7** | 0.1 | 0.2 | **1.4** | 0.3 | 0.2 | **5.2** |
| 18:2n-6 | 0.2 | 0.1 | **1.6** | 0.2 | 0.9 | **1.7** | 0.1 | 0.2 | 0.9 | 0.3 | 0.2 | **3.5** |
| 18:3n-6 | 0.0 | 0.0 | **1.0** | 0.0 | 0.1 | **1.0** | 0.0 | 0.0 | 0.5 | 0.0 | 0.0 | **1.8** |
| 18:3n-3 | 0.0 | 0.0 | **1.1** | 0.1 | 0.1 | **1.2** | 0.0 | 0.0 | 0.6 | 0.1 | 0.0 | **2.3** |
| 18:4n-3 | 0.0 | 0.0 | 0.3 | 0.0 | 0.0 | 0.3 | 0.0 | 0.0 | 0.1 | 0.0 | 0.0 | 0.6 |
| 20:2n-6 | 0.0 | 0.0 | **3.9** | 0.1 | 0.1 | **5.5** | 0.0 | 0.0 | **2.6** | 0.1 | 0.0 | **9.6** |
| 20:3n-6 | 0.1 | 0.0 | **1.5** | 0.1 | 0.5 | **1.4** | 0.0 | 0.0 | 0.9 | 0.1 | 0.1 | **2.9** |
| 20:3n-3 | 0.2 | 0.0 | 0.9 | 0.4 | 0.2 | 0.9 | 0.1 | 0.0 | 0.5 | 0.4 | 0.0 | **1.8** |
| 20:4n-6 | 0.0 | 0.0 | 0.5 | 0.1 | 0.1 | 0.6 | 0.0 | 0.0 | 0.3 | 0.1 | 0.0 | **1.1** |
| 20:4n-3 | 0.0 | 0.0 | 0.6 | 0.1 | 0.0 | 0.6 | 0.0 | 0.0 | 0.3 | 0.1 | 0.0 | **1.2** |
| 20:5n-3 | 0.0 | 0.0 | 0.6 | 0.0 | 0.0 | 0.7 | 0.0 | 0.0 | 0.3 | 0.0 | 0.0 | **1.4** |
| 22:2n-6 | 0.2 | 0.1 | **7.3** | 0.5 | 0.4 | **8.9** | 0.1 | 0.0 | **5.1** | 0.4 | 0.2 | **17.2** |
| 22:6n-3 | 0.0 | 0.0 | 0.1 | 0.0 | 0.0 | 0.6 | 0.0 | 0.0 | 0.3 | 0.0 | 0.0 | **1.2** |
| 24:1n-9 | 0.1 | 0.1 | 0.5 | 0.0 | 0.2 | 0.2 | 0.0 | 0.0 | 0.1 | 0.0 | 0.1 | **2.7** |
|  |  |  |  |  |  |  |  |  |  |  |  |  |
| PUFA | 0.0 | 0.0 | 0.7 | 0.1 | 0.2 | 0.8 | 0.0 | 0.0 | 0.4 | 0.1 | 0.0 | **1.7** |
| n-3 total | 0.0 | 0.0 | 0.5 | 0.0 | 0.0 | 0.6 | 0.0 | 0.0 | 0.3 | 0.0 | 0.0 | **1.3** |
| n-6 total | 0.1 | 0.1 | **1.6** | 0.1 | 0.5 | **1.8** | 0.1 | 0.1 | 0.9 | 0.2 | 0.1 | **3.6** |

Table S3


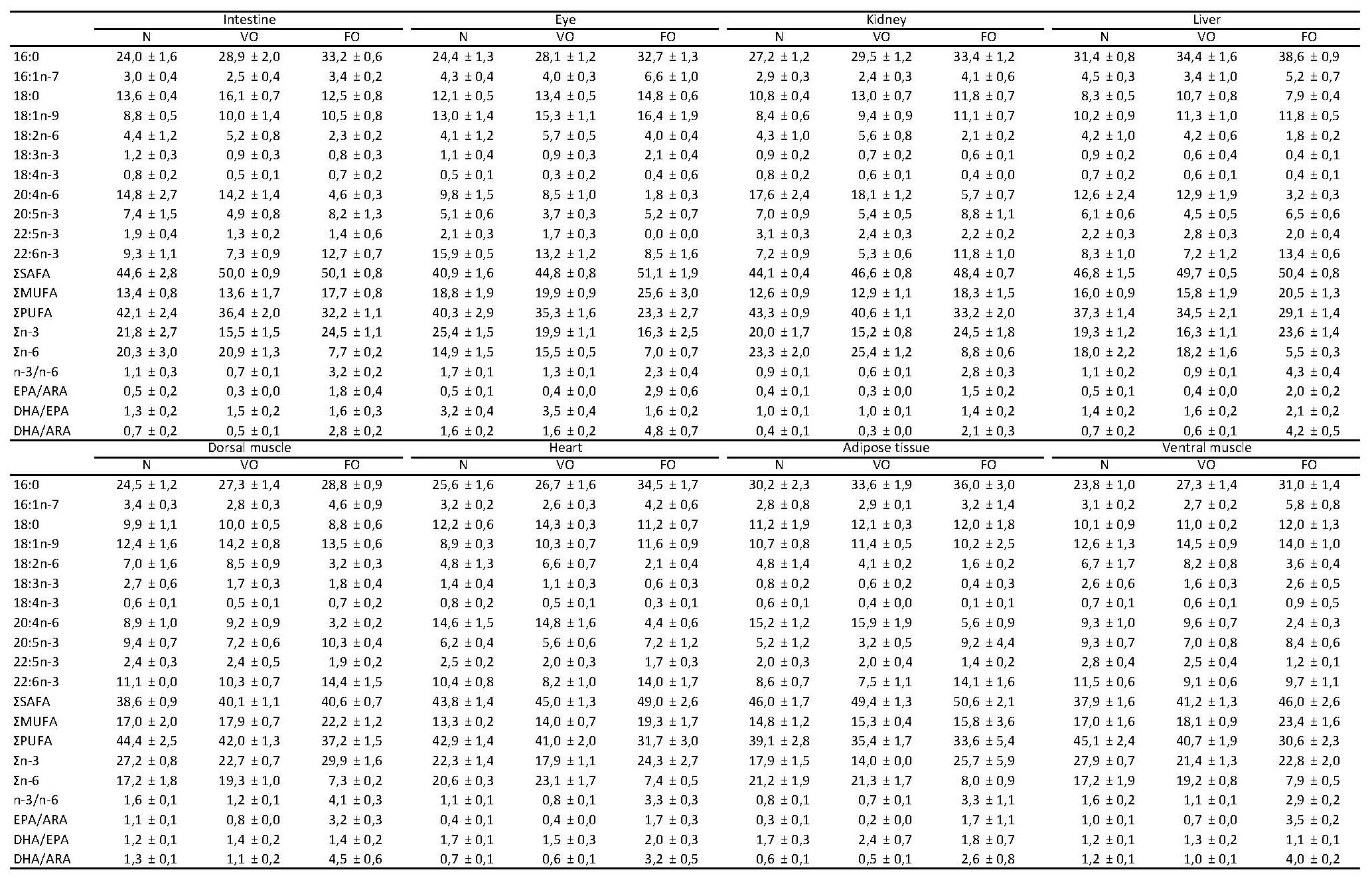


Table S4


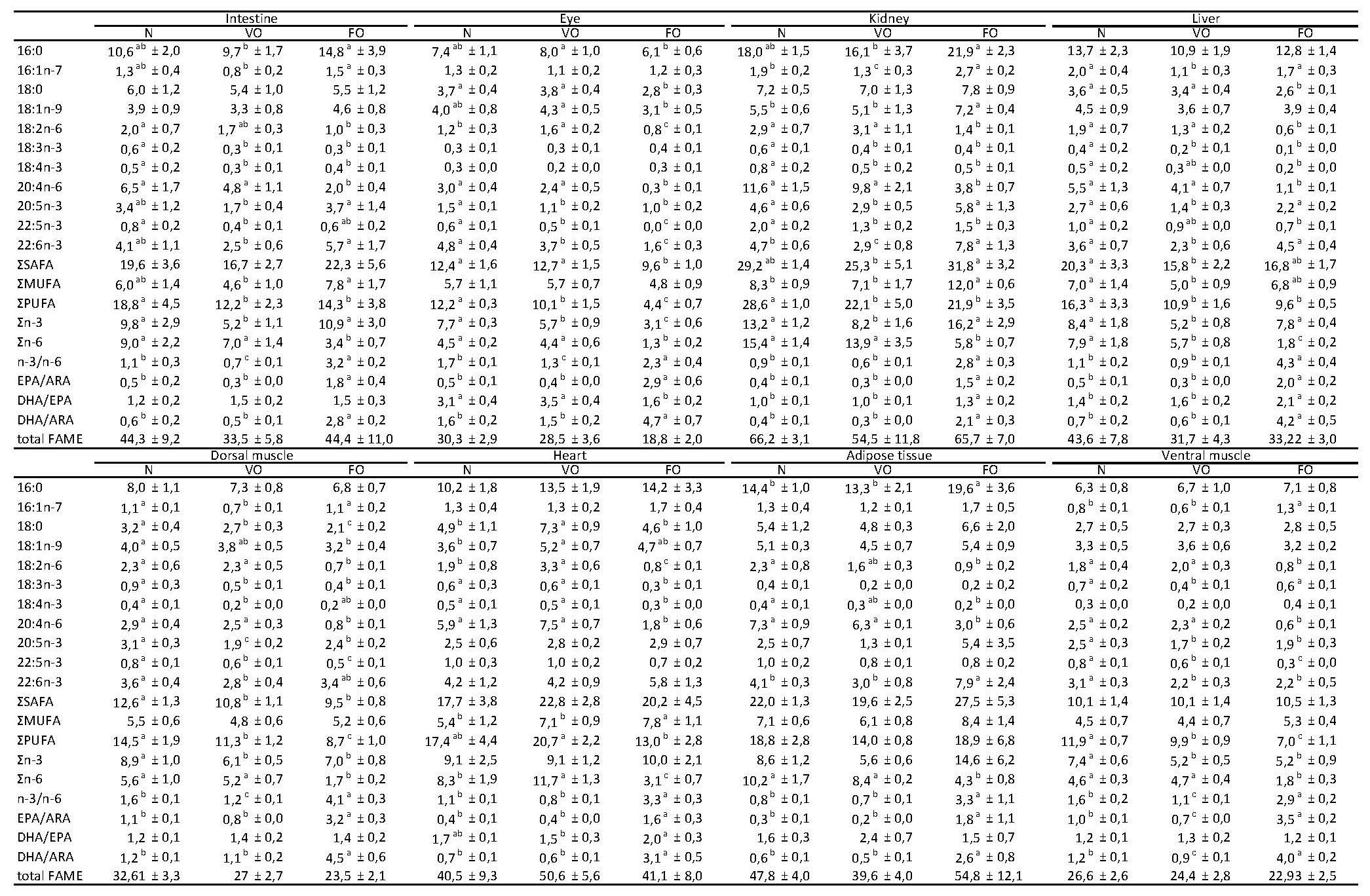


Table S5


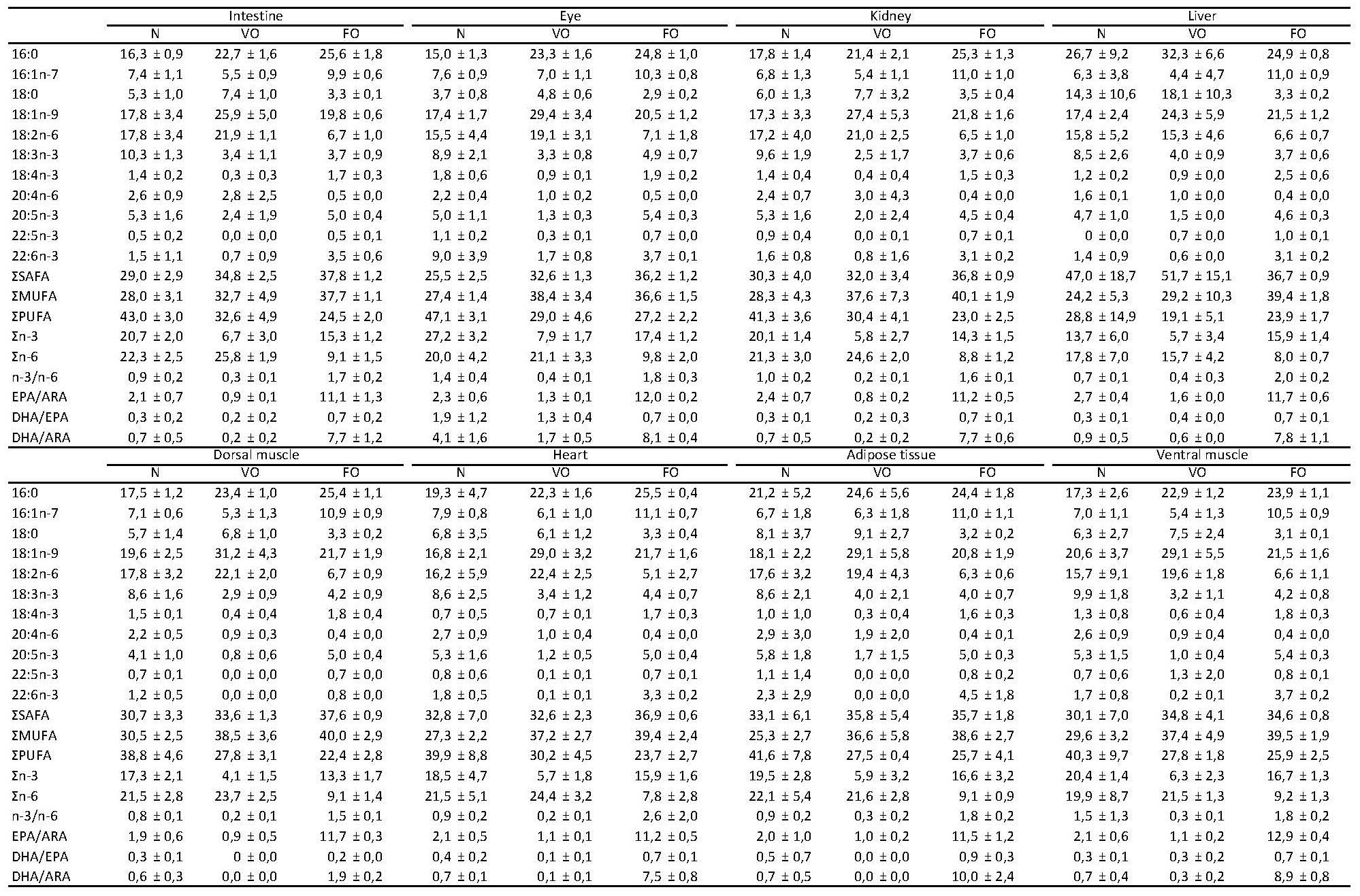


Table S6


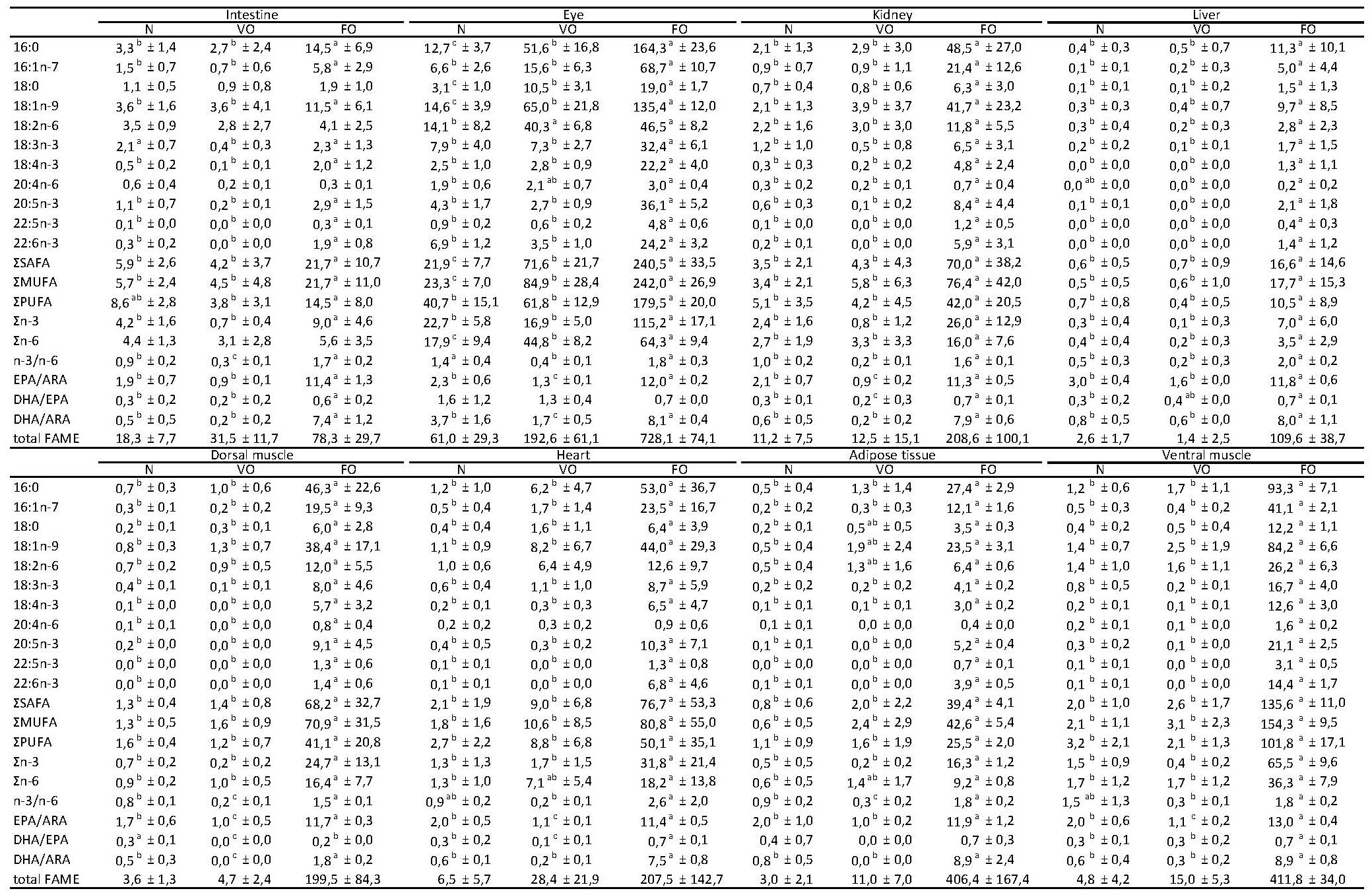

Supplement: File S1 — Table S1. Total lipid mass ratios in tissues of carp after feeding for 210 d on pond zooplankton (N) and zooplankton plus additional feeds containing vegetable oils (VO), and marine fish oils (FO). Table S2. Fatty acid accumulation factors calculated as the quotients of, a) PLFA, and, b) NLFA in carp tissues and food sources (N = pond zooplankton; VO = zooplankton and additional feeds containing vegetable oil; FO = zooplankton and additional feeds containing fish oil). Fatty acids from different food sources in carp were assessed by mixing models using stable isotope analysis of food sources and carp (see text). Fatty acid accumulation in carp tissues is indicated with factors >1 (in bold). Table S3. Relative (%) polar lipid fatty acid (PLFA) composition of common carp tissues ( Cyprinus carpio ) exposed to pond zooplankton (N) as well as on additional meals containing vegetable (VO) and fish (FO) oils (% of total FAME, mean ± SD, n = 5). Table S4. Polar lipid fatty acid (PLFA) mass ratios (mg g dry weight−1) of common carp tissues ( Cyprinus carpio ) exposed to pond zooplankton (N) as well as to additional meals containing vegetable (VO) and fish (FO) oils (% of total FAME, mean ± SD, n = 5). Table S5. Relative (%) neutral lipid fatty acid (NLFA) composition of common carp tissues ( Cyprinus carpio ) exposed to pond zooplankton (N) as well as to additional meals containing vegetable (VO) and fish (FO) oils (% of total FAME, mean ± SD, n = 5). Table S6. Neutral lipid fatty acid (NLFA) mass ratios (mg g dry weight−1) of common carp tissues ( Cyprinus carpio ) exposed to pond zooplankton (N) as well as to additional meals containing vegetable (VO) and fish (FO) oils (% of total FAME, mean ± SD, n = 5). (DOCX) [file pone.0094759.s001.docx]
